# Primary Cilia in Glial Cells: An Oasis in the Journey to Overcoming Neurodegenerative Diseases

**DOI:** 10.3389/fnins.2021.736888

**Published:** 2021-09-30

**Authors:** Soo Mi Ki, Hui Su Jeong, Ji Eun Lee

**Affiliations:** ^1^Department of Health Sciences and Technology, Samsung Advanced Institute for Health Sciences and Technology, Sungkyunkwan University, Seoul, South Korea; ^2^Samsung Medical Center, Samsung Biomedical Research Institute, Seoul, South Korea

**Keywords:** glia, primary cilia, myelination, nerve regeneration, neurological disease

## Abstract

Many neurodegenerative diseases have been associated with defects in primary cilia, which are cellular organelles involved in diverse cellular processes and homeostasis. Several types of glial cells in both the central and peripheral nervous systems not only support the development and function of neurons but also play significant roles in the mechanisms of neurological disease. Nevertheless, most studies have focused on investigating the role of primary cilia in neurons. Accordingly, the interest of recent studies has expanded to elucidate the role of primary cilia in glial cells. Correspondingly, several reports have added to the growing evidence that most glial cells have primary cilia and that impairment of cilia leads to neurodegenerative diseases. In this review, we aimed to understand the regulatory mechanisms of cilia formation and the disease-related functions of cilia, which are common or specific to each glial cell. Moreover, we have paid close attention to the signal transduction and pathological mechanisms mediated by glia cilia in representative neurodegenerative diseases. Finally, we expect that this field of research will clarify the mechanisms involved in the formation and function of glial cilia to provide novel insights and ideas for the treatment of neurodegenerative diseases in the future.

## Introduction

Since the identification of primary cilium, a cellular organelle composed of microtubules, in a variety of cells, including rabbit kidney tubular epithelial cells, human pancreatic ductal epithelial cells, seminal vesicle epithelial cells, uterine fundus epithelial cells, and thyroid gland epithelial cells, about a century ago (Bloodgood, 2009), extensive research continues to elucidate its functional significance. Based on previous findings, primary cilia are believed to serve as major hubs of various signaling pathways such as Wnt, Shh, Notch, PDGF, and mTOR in most cells (Wheway et al., 2018). Given that these signal transductions are involved in pivotal developmental processes, including cell proliferation, migration, and differentiation (Wong et al., 2001; Basson, 2012; Duronio and Xiong, 2013), impairment of primary cilia is closely associated with many diseases (Wheway et al., 2018). In particular, several neurodevelopmental diseases, such as Joubert syndrome, Bardet–Biedl syndrome, and Meckel–Gruber syndrome are representative ciliopathies (Rooryck et al., 2007; Giordano et al., 2009; Hildebrandt et al., 2009).

The tight link between the primary cilia and nervous system-related diseases has prompted researchers to investigate the role of cilia in neurons. It is noteworthy that several previous reports have suggested that primary cilia of neurons are important for Shh signaling-mediated cell proliferation in postnatal development of the hippocampus and the cerebellum (Breunig et al., 2008; Han et al., 2008; Spassky et al., 2008). However, most studies have been limited to adult neuronal cilia in the central nervous system (CNS) (Kirschen and Xiong, 2017; Park S.M. et al., 2019). Indeed, it has long been observed that primary cilia are present not only in mature neurons but also in several kinds of cells, including neural crest cells, neural progenitor cells, and glial cells (Etchevers et al., 2019; Park S.M. et al., 2019; Yusifov et al., 2021). Moreover, primary cilia are organelles that undergo assembly and disassembly depending on the type of cell or developmental processes. Therefore, intensive studies on non-neuronal cilia have been emphasized to comprehensively understand the function of primary cilia in the nervous system.

Glial cells are the main components of the nervous system; they are roughly equal to the number of neurons and play important functional and structural roles in supporting neurons during development and regeneration. There are several types of glial cells both in the CNS and the peripheral nervous system (PNS): astrocytes, oligodendrocytes (OLGs), and microglial cells in the CNS and Schwann cells, satellite glial cells, enteric glial cells, and olfactory ensheathing cells in the PNS (Compston et al., 1997; Jessen and Mirsky, 2005; Domingues et al., 2016). Indeed, the presence of primary cilia has already been observed in astrocytes and OLGs of the CNS and in Schwann cells of the PNS, although studies on the role of glia cilia are in elementary stages (Bishop et al., 2007; Kasahara et al., 2014; Falcon-Urrutia et al., 2015).

Several neuropathies with defective axonal regeneration result from glial cell dysfunction (Lehmann and Hoke, 2010; Gritsch et al., 2014; Li et al., 2019), and glial impairment due to nerve injury leads to inflammatory neuropathy, which affects myelination (Willison and Yuki, 2002). Many studies have attempted to treat demyelination-associated diseases in a variety of ways, including immune suppression using steroids (Rahmlow and Kantarci, 2013; Klein et al., 2019; Gwathmey and Grogan, 2020); however, there have been no targeted therapies developed till date. Recent studies have reported that OLGs progenitor cells (OPCs) derived from neural stem cells are effective in regenerating damaged axons through myelination activation (Murphy and Franklin, 2017) and that astrocytes derived from embryonic stem cells are effective in protecting neurons from neurotoxicity and death (Barbeito, 2018). This suggests that therapies focusing on the function of glial cells could be an alternative treatment for neuropathy. Accordingly, in this review, we will address some recent findings regarding the importance of targeting glial cells, especially primary cilia of glia, for the treatment of developmental and degenerative diseases of the nervous system (Table 1).

**TABLE 1:** Role of glial primary cilia in the pathogenesis of neurodegenerative diseases.

Glial cells	Diseases	Key molecules	Role in primary cilia	References
Astrocyte	Alzheimer’s disease (AD)	GPR37L1, PTCH1	• Accumulated Aβ shortens primary cilia and inhibits cilia assembly • Impaired primary cilia suppress Shh activation	Vorobyeva and Saunders, 2018; La Sala et al., 2020
	Parkinson’s disease (PD)	LRRK2, RAB10, CP110, RILPL1	• Mutated LRRK2 inhibits primary cilia assembly by interfering with a removal of CP110 from the ciliary base • Mutated LRRK2 induces an interaction of RILPL1 and RAB10 and disrupts Shh signaling by inhibiting cilia assembly	Steger et al., 2017; Dhekne et al., 2018; Sobu et al., 2021
	Huntington’s disease (HD)	HTT, HAP1, PCM1	• Mutated HTT induces accumulation of PCM1 in the ciliary base, leading to abnormal elongation of cilia • The abnormally elongated cilia activates Wnt signaling	Keryer et al., 2011; Lancaster et al., 2011
Oligodendrocyte progenitor cell (OPC)/Oligodendrocyte (OLG)	Multiple sclerosis	SMO, GLI1	• Primary cilia-mediated Shh activation increases expression of *Gli1* and *Smo*, which are associated with remyelination	Ferent et al., 2013; Ortega et al., 2013
	Leukodystrophy	TUBB4A	• Mutated TUBB4A inhibits cilia assembly and Shh signal transduction by affecting microtubule dynamics	Simons et al., 2013; Curiel et al., 2017
Schwann cell	Amyotrophic Lateral Sclerosis (ALS)	SOD1, ACIII, ACTIN, WNT3A, β-catenin	• Mutated SOD1 reduces primary cilia in neurons and Schwann cells with increased Actin and Wnt genes	Ma et al., 2011; Tawk et al., 2011; de Oliveira et al., 2013; Yu et al., 2013; Lee, 2020
	Charcot Marie Tooth disease (CMT)	PMP22, MPZ, EGR2, ACIII, cAMP, SMO, PTCH1, WNT1, WNT3A, WNT5A, β-catenin, YAP/TAZ	• Down-regulation of cAMP in neuronal primary cilia can cause demyelination • In addition to cAMP signaling factors, key molecules of Shh, Wnt, and Hippo signaling are present in Schwann cell cilia • These signaling pathways are involved in ciliogenesis and (re)myelination after nerve injury	LeBlanc et al., 1992; Hattori et al., 2003; Morgello et al., 2004; Vandevelde and Zurbriggen, 2005; Wang et al., 2011; Yoshimura and Takeda, 2012; G et al., 2014; Lempp et al., 2014; Qiu et al., 2016
	Human Immunodeficiency Virus-associated distal sensory polyneuropathy (HIV-SN)			
	Canine distemper virus demyelinating leukoencephalitis (CDV-DL)			
	Diabetic neuropathy	IP_3_, IP_3_R, P2Y2, P2RY2,	• Ca^2+^ signaling regulators are associated with ciliogenesis of Schwann cells • Diabetic stress inhibits Ca^2+^ signaling and leads to dysfunction of Schwann cells	Hatayama et al., 2011; Wann et al., 2012; Weisman et al., 2012; Ino et al., 2015; Goncalves et al., 2018
	Lewy body pathology	PSA, NCAM, α–synuclein	• PSA-NCAM complex exists in glia cilia and its decrease is associated with ciliary defects • Decrease of the PSA-NCAM complex causes aggregation of α–synuclein, leading to Lewy body pathology	Han et al., 2008; Iqbal et al., 2020; Matsumoto et al., 2019; Papastefanaki et al., 2007; Sorrentino et al., 2019

## Primary Cilium in Astrocytes: A Key Modulator of Neuronal Signaling in the Central Nervous System

Astrocytes, the most abundant glial cells in the CNS, are important for the formation and maintenance of the blood-brain barrier to secrete a variety of neurotrophic factors during synaptogenesis, neuronal differentiation, and neuronal survival (Carson et al., 2006; Fakhoury, 2018). In neurodegenerative diseases, astrocytes elevate the production of cytotoxic molecules, including reactive oxygen species (ROS), and inflammatory mediators, including COX2, IL-1β, TNF-α, and IL-6 (Akiyama et al., 2000; McGeer and McGeer, 2002). Moreover, in inflammatory conditions, astrocytes affect surrounding cells in response to pro-inflammatory cytokines/chemokines and nitric oxide secreted by immune cells present in the CNS (Rothhammer and Quintana, 2015; Sofroniew, 2015). For example, the pro-inflammatory factors, whose production is increased by astrocytes, induce apoptosis of neighboring neurons (Simi et al., 2007; McCoy and Tansey, 2008), and this leads to several CNS neurodegenerative diseases, such as Alzheimer’s disease (AD), Parkinson’s disease (PD), and Huntington’s disease (HD) (Habib et al., 2020).

**FIGURE 1:**
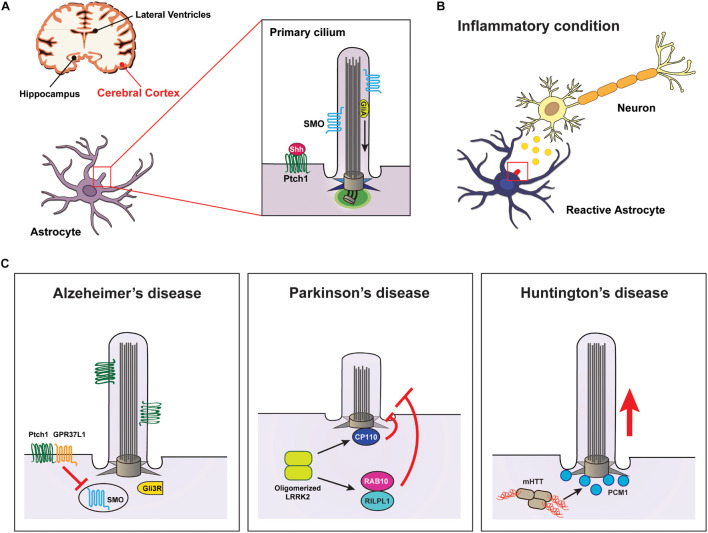
Signaling through the primary cilia in astrocytes is associated with neurodegenerative diseases. **(A)** Diagram showing the primary cilia of astrocytes associated with diseases occurring in the cerebral cortex. **(B)** Neuroinflammation affects Shh signal transduction through the primary cilia in astrocytes. **(C)** Examples of neurodegenerative diseases involved in impairments of primary cilia in astrocytes. Elevated Aβ attenuates Shh signaling in astrocyte cilia, leading to Alzheimer’s disease. Defects in LRRK2 cause Parkinson’s disease by inhibiting astrocyte cilia formation by preventing the removal of CP110 from the ciliary base or by inducing a complex of RILPL1 and RAB10. Huntington’s disease results from mutated HTT-induced PCM1 accumulation at the ciliary base of astrocytes.

Observations of primary cilia in astrocytes (Bishop et al., 2007; Danilov et al., 2009; Yoshimura et al., 2011; Kasahara et al., 2014) and the identification of Shh signaling molecules such as smoothened (SMO) and patched 1 receptor (PTCH1) in the cilia of astrocytes (Yoshimura et al., 2011) have led to an understanding of the role of astrocyte cilia in CNS development and diseases (Figure 1). Several previous studies have suggested that astrocyte cilia may play an important role in Shh signaling to control cellular biological processes in inflammatory conditions (Amankulor et al., 2009; Jin et al., 2015; Allahyari et al., 2019); however, only little direct evidence related to this finding has been reported. In order to explain recent insights related to the role of astrocyte cilia in inflammatory CNS diseases, we cover several experimental results that suggest a direct involvement of astrocyte cilia in some representative neurodegenerative diseases in this review.

### Alzheimer’s Disease

Alzheimer’s disease is clinically characterized by impaired memory and cognitive function due to a gradual loss of neurons. The pathological hallmark of AD is the extracellular plaque deposition of insoluble amyloid-beta (Aβ) peptide, a flame-shaped neurofibrillary tangle (NFT) of the microtubule-binding protein TAU, which induces neuroinflammation in the brain (Fakhoury, 2018). Recent findings have suggested that astrocytes play a role in promoting neurodegenerative processes in AD by increasing neurotoxicity via secretion of inflammatory cytokines under activation of Aβ (De Strooper and Karran, 2016; Liao et al., 2016; Keren-Shaul et al., 2017). Notably, increased Aβ deposition affects the assembly and length control of primary cilia in astrocytes, thereby inhibiting the activation of the Shh signaling pathway and eventually disrupting neuronal survival (Vorobyeva and Saunders, 2018).

The G protein-coupled receptor 37-like 1 (GPR37L1) is expressed in cerebellar Bergmann glia (BG) astrocytes specifically, and it is involved in the proliferation and differentiation of cerebellar granule neurons and the maturation of Purkinje neurons (Valdenaire et al., 1998; Meyer et al., 2013). Previous studies have reported that murine Gpr37l1 physically interacts with Ptch1 in the periciliary membrane of BG cells (Marazziti et al., 2013). Moreover, a recent study found that GPR37L1 binds to PTCH1 to regulate Shh signal transduction by inducing the ciliary translocation of SMO in cerebellar astrocytes (La Sala et al., 2020; Figure 1C and Table 1).

In mice AD models, particularly, serotonin receptors of type 6 (5-HT_6_) regulates neuronal cilia length, morphology, and composition that are related to cognition (Brodsky et al., 2017; Hu et al., 2017). 5-HT_6_ is also expressed in astrocytes and localized in the primary cilia (Hirst et al., 1997; Jiang et al., 2019; Barbeito et al., 2021). The binding of serotonin to 5-HT_6_ activates the Shh pathway by stimulating GPCR-dependent cAMP signaling (Jiang et al., 2019).

Astrocytes from mice with nervous system injury exhibited a disturbance of Shh signaling in the vicinity of the lesion site in a study (Allahyari et al., 2019). Intriguingly, activation of Shh signaling also affects the proliferation of reactive astrocytes under chronic injury conditions (Sirko et al., 2013). Additionally, activated Shh signaling inhibits inflammation by limiting leukocyte invasion under injury conditions (Allahyari et al., 2019). It is notable that astrocytes responding to Shh signals affect not only themselves but also neurons as they reduce the expression of GFAP in neurons to promote neuroprotection (Sirko et al., 2013; Ugbode et al., 2017). Furthermore, Shh signaling activated in neurons acts as a physiological cue for astrocytes (Amankulor et al., 2009; Farmer et al., 2016). Thus, these findings suggest the importance of Shh signal transduction in communication between neurons and astrocytes in the CNS, particularly in injury-induced neurotoxic inflammatory states. Taken together, these studies suggest that primary cilia in astrocytes are pivotal in regulating Shh signaling to facilitate proliferation and maturation of neural stem and progenitor cells to repair the damaged CNS, including AD.

### Parkinson’s Disease

Parkinson’s disease is a neurodegenerative disease characterized by the degeneration of ventral midbrain dopaminergic (DA) neurons and the accumulation of α-synuclein-positive cytoplasmic inclusions in neurons (Dauer and Przedborski, 2003; Surmeier, 2018). Although PD has been largely regarded as a DA neuronal disease, recent studies have indicated the involvement of astrocytes; in particular, some PD-related genes appear to play important roles in several astrocyte functions, such as the control of inflammation, autophagy, calcium signaling, and neuroprotection (Hernandez et al., 2016; Booth et al., 2017). For instance, mutations in leucine-rich repeat kinase 2 (*LRRK2*), the most frequent causative gene identified in PD (Zimprich et al., 2004; Trinh et al., 2014), result in dysfunction of astrocytes in the clearance of α-synuclein, thereby decreasing the number of DA neurons (Greggio et al., 2006). LRRK2 is a multifunctional protein comprising several domains at its C-terminus, including a leucine-rich domain (LRR), a GTPase domain, the carboxy-terminal region of the Ras domain, a kinase domain, and a WD40 domain (Zimprich et al., 2004; Greggio and Cookson, 2009). LRRK2 is constitutively expressed in most of cells in the nervous system, including neurons, astrocytes, microglia, and OLGs (Miklossy et al., 2006). However, most LRRK2-related studies on PD pathogenesis have been limited in neurons. Loss of LRRK2 in cortical neurons causes mitochondrial dysfunction due to decreased Ca^2+^ extrusion of Na^+^/Ca^2+^/Li^+^ exchanger (NCLX) efflux (Ludtmann et al., 2019) and reduction in glutamatergic transmission and synaptic protein expression (Beccano-Kelly et al., 2014). In addition, in PD, *LRRK2* mutations induce cell death by activating kinase activity (Greggio et al., 2006) or microtubule-associated neurotoxicity and neurodegeneration by oligomerizing LRRK2 (Kett et al., 2012; Dhekne et al., 2018; Watanabe et al., 2020).

Based on the function of LRRK in controlling microtubule dynamics through direct interaction with microtubules (Gillardon, 2009; Kett et al., 2012), a potential role for LRRK2 in ciliary biogenesis has been suggested. Several studies have reported the key roles of LRRK2 in ciliogenesis: LRRK2 is involved in the removal of CP110 and the recruitment of TTBK2 at the mother centriole (the base of cilium) (Sobu et al., 2021). It is also involved in the phosphorylation of RAB10 on binding to RILPL1 (Steger et al., 2017; Dhekne et al., 2018; Figure 1C and Table 1). In addition, the data that LRRK2 interacts with microtubule components, such as TUBB, and affects microtubule acetylation (Law et al., 2014) suggest that LRRK2 may be involved in axonemal tubulin acetylation for cilia assembly. Thus, these previous findings, including that inhibition of Shh leads to neuronal damage by interfering with neuroprotection against neurotoxicity (Patel et al., 2017), collectively suggest that mechanisms regulating primary cilia generation in astrocytes may be a target for the treatment of neurodegenerative diseases such as PD.

### Huntington’s Disease

Huntington’s disease, the most common autosomal dominant neurodegenerative disease, presents with pathological features such as neuronal dysfunction associated with excessive movement and cognitive impairment. It is well known that CAG triplet repeat expansion, encoding polyglutamine, within huntingtin (HTT) leads to the production of mutant HTT (mHTT) fragments, which is a major cause of HD (Finkbeiner, 2011). Previous study findings, including that mHTT is specifically expressed in astrocytes in a mouse model of HD (Bradford et al., 2009), have suggested that astrocytes are critically involved in the pathological mechanism of HD (Diaz-Castro et al., 2019; Gray, 2019). Remarkably, a recent study reported that a reduction of mHTT in astrocytes in an HD mouse model resulted in recovery of neuron functions, including neuroprotection (Wood et al., 2019). However, the molecular mechanisms underlying the pathology of HD in astrocytes remain poorly understood. The interaction of HTT with microtubules regulates microtubule-dependent transport for cilia formation by binding to huntingtin-associated protein 1 (HAP1) and pericentriolar material 1 (PCM1) proteins (Keryer et al., 2011; Table 1). Accordingly, mHTT induces the accumulation of PCM1 around the centrosomes, leading to the formation of abnormally long cilia that might be involved in inhibiting neuroprotection (Keryer et al., 2011; Figure 1C).

Previous studies have shown that HAP1 interacts with Abelson helper integration site 1, which plays a role in primary cilia-mediated Wnt signal transduction (Sheng et al., 2008; Lancaster et al., 2011), and that mHTT induces the accumulation of cytoplasmic β-catenin (Godin et al., 2010; Ghatak and Raha, 2018). In addition, in the process of ciliogenesis, mHTT affects membrane trafficking by interfering with the activity of small GTPases, such as RAB8 and RAB11 (Li et al., 2009, 2010; Knodler et al., 2010). Taken together, these data suggest that HTT may be essential for neuroprotection-related Wnt signaling by regulating primary cilia assembly through activation of RAB8 and RAB11 in astrocytes, and they also suggest that abnormalities of astrocyte cilia may be closely associated with HD pathogenesis.

### Potential Role of Astrocyte Cilia in NF-κB Signaling

The role of primary cilia in astrocytes, which are glial cells involved in various processes such as neuronal support, homeostasis maintenance, and neuronal transmission, remains unclear in neurodegenerative diseases. However, the findings that astrocytes have primary cilia and multiple neurological disease mechanisms are associated with ciliary functions have generated immense interest in the role of astrocyte cilia. Several inflammation-related signaling pathways, including the Shh, Wnt, and NF-κB signaling pathways, are controlled by primary cilia (Wann and Knight, 2012; Baek et al., 2017). Thus, previous studies have reported an involvement of primary cilia in many inflammatory diseases such as obesity, polycystic kidney disease, and osteoarthritis (Song et al., 2017; Ritter et al., 2018; Sheffield et al., 2018). Although the role of primary cilia in inflammatory signaling-related neurological diseases has not been much investigated, it has been reported that the induction of inflammation by lipopolysaccharide treatment shortens the length of primary cilia in hippocampal neurons (Baek et al., 2017).

Persistent activation of NF-κB, which is considered a master regulator of inflammation (Hayden and Ghosh, 2012), is associated with several neurodegenerative diseases including AD, PD, and ALS (Ju Hwang et al., 2019; Singh et al., 2020; Kallstig et al., 2021). Based on the important role of astrocytes in the regulation of neuroinflammation (Colombo and Farina, 2016), studies on the mechanism of NF-κB signaling regulation in astrocytes have gained interest. Previous studies have revealed that inactivation of NF-κB in astrocytes reduces the expression of chemokines in mice with spinal cord injury (Brambilla et al., 2005) and decreases demyelination in mice with vascular dementia (Saggu et al., 2016). These findings suggest the importance of the inhibitory regulation of NF-κB signaling in astrocytes to prevent neurodegenerative diseases.

Notably, in a mouse model, activation of NF-κB signaling specific for astrocytes not only induced the secretion of several chemokines but also impaired ependymal ciliary formation (Lattke et al., 2012). Further studies have revealed that primary cilia of fibroblasts and chondrocytes in response to inflammation are involved in NF-κB signaling through IKK activity (Wann and Knight, 2012; Wann et al., 2014). However, direct evidence for the effect of NF-κB signaling regulation on ciliary formation in astrocytes is lacking. Nevertheless, previous findings suggest the importance of primary cilia of astrocytes in regulating NF-κB signaling to prevent or treat inflammation-associated neurodegenerative diseases.

## Primary Cilium in Oligodendrocytes: A Key Mediator for Myelination in the Central Nervous System

**FIGURE 2:**
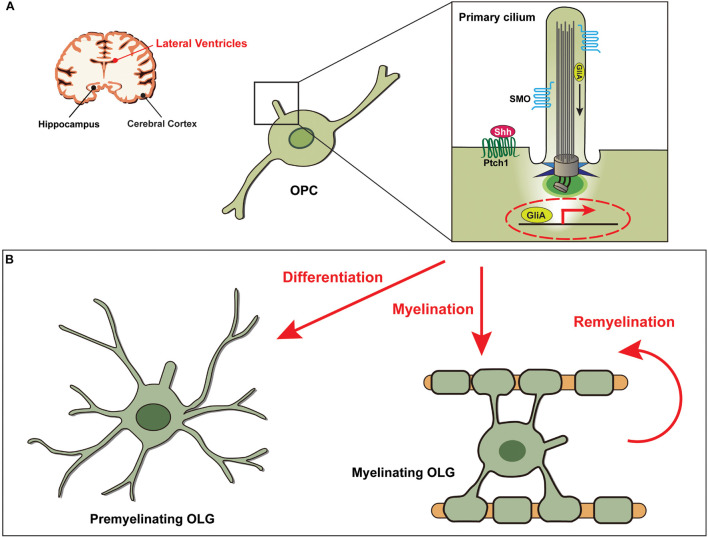
Shh signaling through the primary cilia in OPCs is essential for the development of oligodendrocytes. **(A)** Diagram showing the primary cilia of oligodendrocyte progenitor cells (OPCs) associated with functions in the lateral ventricles. **(B)** Shh signaling is activated by binding of the Shh ligand to the Ptch1 receptor, leading to the release of SMO from Ptch1 and its translocation into the primary ciliary axoneme. GliA, the activator form of Gli, then induces transcription of Shh target genes, which are involved in differentiation of OPCs to oligodendrocytes (OLGs) and in (re)myelination of OLGs.

OLGs differentiated from OPCs are involved in CNS myelination, a process that wraps around nerve axons by producing multiple layers of myelin that extend from the cell membrane (Nave, 2010; Stadelmann et al., 2019). These cells are susceptible to cytotoxicity/excitotoxicity, and defects in OLGs result in several neurological diseases, including AD, multiple sclerosis (MS), and schizophrenia (Kuhn et al., 2019). Impairment of the OLG differentiation process is associated with aging and the pathogenesis of MS (Tiane et al., 2019). The role of primary cilia in the pathological mechanisms of demyelinating diseases has not been investigated much, but recent studies have reported that major ciliary molecules, such as kinesin family member 3A (KIF3A) and intraflagellar transport 81, and Shh signaling components, such as SMO and PTCH1, are expressed in mouse cortical OPCs (Cahoy et al., 2008; Zhang et al., 2014; Figure 2A). It was further found that primary cilia of OPCs were disassembled during differentiation into OLGs (Falcon-Urrutia et al., 2015). Notably, in a mouse model, depletion of *KIF3A* in OPCs inhibited OLG proliferation and differentiation by affecting cilia-dependent Shh signaling and subsequently induced motor defects due to a decrease in myelinated axons (Cullen et al., 2021). Interestingly, the abnormal length of primary cilia in OPCs led to impairment of OLG differentiation (Dugas et al., 2010; Hudish et al., 2016). Therefore, control of ciliary biogenesis, including assembly and disassembly, during OLG differentiation could be a potential target for the treatment of demyelinating disorders.

### Multiple Sclerosis

Multiple sclerosis is a chronic inflammation-induced demyelinating disease that results in progressive neurological impairment in the CNS. As the CNS is damaged, the OPCs in the injured regions differentiate into OLGs. If this mechanism is disturbed, less OLGs are produced, resulting in MS (Dulamea, 2017; Kuhn et al., 2019). Hence, previous studies have suggested that the control of remyelination by differentiated OLGs during neuronal protection/regeneration is pivotal in the treatment of MS (Stangel et al., 2017; Baldassari et al., 2019). Accordingly, several therapeutic candidate targets, which are mainly involved in the development or myelination of OLGs via Shh, Wnt, and LINGO1 signaling, have been proposed (Fancy et al., 2009; Cole et al., 2017; Ineichen et al., 2017).

In particular, attention has been paid to Shh signal transduction since it is a key trigger for regulating the differentiation and remyelination by OLGs (Thomas et al., 2014; Traiffort et al., 2016; Sanchez and Armstrong, 2018). In the process of remyelination, the expression of Shh signaling-associated molecules, including SMO and GLI1, is increased, and Shh treatment induces the generation of OPCs and OLGs (Ferent et al., 2013; Table 1). Furthermore, it has been demonstrated that treatment with Shh at injury sites increased axonal myelination by inducing OPC recruitment into the damaged area (Thomas et al., 2014). It is notable that in a chronically demyelinated corpus callosum, a subpopulation of neural stem cells responding to Shh is able to generate OPCs and, thus, induce remyelination (Sanchez et al., 2018). However, the molecular mechanisms involved in OPC development and OLG remyelination by Shh signaling remain elusive. Thus, further studies to elucidate the principal molecular axes may accelerate the development of potential therapies for MS. Based on the requirement of primary cilia for Shh signal transduction essential for tissue development and homeostasis (Briscoe and Therond, 2013; Anvarian et al., 2019), we assume that primary cilia of OPCs are likely to play a key role during differentiation into OLGs (Figure 2B).

### Leukodystrophies

Leukodystrophies, which refer to disorders with wasting (*dystrophy*) and white matter (*leuko*) in the brain, are heterogeneous neurodegenerative diseases that primarily affect myelination in the CNS (Vanderver et al., 2015). Defects in the development and myelination of OLGs are closely related to the pathogenesis of leukodystrophies (Raymond, 2017). A recent study that performed high-content screening using small molecules with mouse samples suggested that the Shh signaling pathway may be a potential therapeutic target for leukodystrophies (Atzmon et al., 2018). The mechanism by which the compounds work is probably associated with the Shh-dependent myelination of OLGs. In addition, there is a possibility that primary cilia of OLGs are involved in Shh signaling for the regulation of myelination (Figure 2B).

The identification of mutations in tubulin beta class IVA (*TUBB4A*) in hypomyelination with atrophy of the basal ganglia and cerebellum, a type of leukodystrophy, prompted us to elucidate the role of TUBB4A in OLGs (van der Knaap et al., 2002). Recent studies using cell type-specific RNA-sequencing analyses have shown that *TUBB4A* is highly expressed in OLGs compared to that in neurons, astrocytes, and microglia in the CNS (Zhang et al., 2014). Corresponding to the involvement of TUBB4A, an isoform of β-tubulin, in microtubule assembly (Simons et al., 2013), mutations in *TUBB4A* lead to disruption of microtubule stability and polymerization in OLGs (Curiel et al., 2017; Table 1). In addition, mice with mutations in *TUBB4A* develop severe perturbation of myelin due to microtubule abnormalities in OLGs, resulting hypomyelination and demyelination (Duncan et al., 2017). Although the essential roles of microtubules in various cellular functions, including the development of OLGs, are well known (Lunn et al., 1997; Song et al., 2001; Zuchero et al., 2015), the TUBB4A-mediated mechanism of myelination remains unclear. Given the role of TUBB4A in microtubule assembly (Tischfield et al., 2011; Simons et al., 2013; Duncan et al., 2017), however, it is possible to speculate that TUBB4A may be involved in the assembly of primary cilia in OLGs. Indeed, other tubulin proteins, such as TUBB4 and TUBB4B act as key regulators by interacting with KIF11 in cilia assembly (van Dam et al., 2019; Janke and Magiera, 2020; Zalenski et al., 2020). Taken together, in the absence of treatments for demyelinating diseases in the CNS, studies investigating the role of primary cilia in microtubule-associated myelination processes will help better understand the disease mechanisms.

## Primary Cilium in Schwann Cells: A Key Signaling Modulator in the Peripheral Nervous System

In the PNS, Schwann cells are counterparts of OLGs in the CNS. There are two types of Schwann cells, myelinating and non-myelinating, both of which interact with axons to regulate the function of surrounding cells, including neurons and muscle cells (Campana, 2007; Barton et al., 2017; Boerboom et al., 2017). While myelinating Schwann cells induce myelination progression in response to several signaling pathways, such as the PI3K/Akt/mTOR, Wnt, and Nrg1/ErbB signaling (Norrmen and Suter, 2013; Boerboom et al., 2017; Torii et al., 2019), non-myelinating Schwann cells primarily respond to ErbB signaling to regulate axonal, muscle, and neuromuscular junction development (Carroll et al., 1997; Garratt et al., 2000; Newbern and Birchmeier, 2010). Damage to Schwann cells thus leads to either demyelinating diseases or neurotrophic diseases along with axonal defects (Boerboom et al., 2017; Park H.T. et al., 2019). In response to nerve injury, Schwann cells participate in nerve regeneration by activation of their features for de-differentiation and reprogramming (Chen et al., 2007; Martini et al., 2008; Jessen and Mirsky, 2016). Moreover, during the repair process of injured nerves, an increase in cytokines induces an immune response through the activation of C-Jun in Schwann cells (Arthur-Farraj et al., 2012).

**FIGURE 3:**
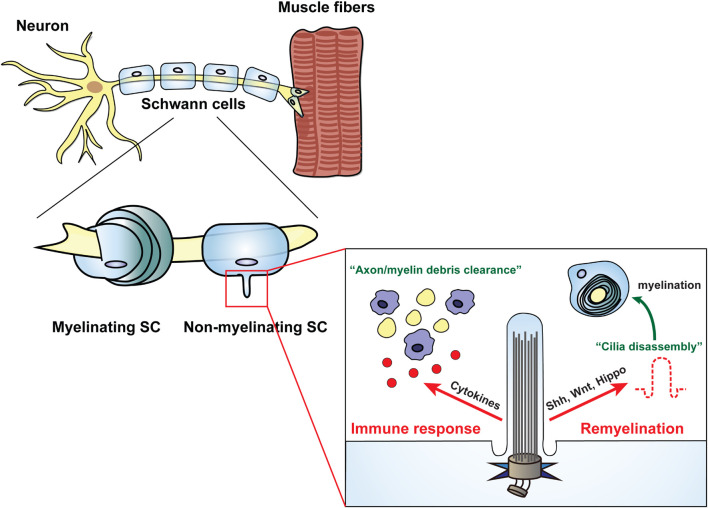
Schwann cells regulate various cellular processes through the primary cilia. Primary cilia generated in non-myelinating Schwann cells are involved in cellular events through dynamic biogenesis of assembly and disassembly. In the immune response state, the primary cilia of Schwann cells induce clearance of axon debris by macrophages through activation of inflammatory cytokines. In PNS injury, Schwann cells play a role in axonal regeneration by inducing remyelination through the disassembly of primary cilia, which is dependent on several signal transductions such as Shh, Wnt, and Hippo.

Since the first identification of primary cilia in the autonomic nerves and ganglia of adult rats in the PNS (Grillo and Palay, 1963), only a few studies on the role of glia cilia have been reported. Although Schwann cells have emerged as a pivotal therapeutic target for PNS disorders (Lehmann and Hoke, 2010; Barton et al., 2017), direct evidence for the role of cilia in Schwann cells in disease-related mechanisms is very limited. The Shh signal pathway is involved in proliferation and myelination during the development of Schwann cells, and more importantly, it is activated to promote nerve regeneration after injury (Hashimoto et al., 2008; Yoshimura and Takeda, 2012). Hence, the findings of studies focusing on the role of primary cilia of Schwann cells in myelination, particularly on their role in related signaling such as Shh, are noteworthy (Yoshimura and Takeda, 2012; Figure 3). In the following sections, we discuss the involvement of Schwann cell cilia in the regulation of nerve regeneration and mechanisms of neurodegenerative diseases and highlight the importance of investigating the functions of Schwann cell cilia in PNS disorders.

### Amyotrophic Lateral Sclerosis

Amyotrophic lateral sclerosis (ALS), characterized by progressive muscle weakness and ultimately fatal loss of muscle function, is a well-known peripheral neurodegenerative disease. It is alternatively called motor neuron disease and is generally diagnosed using nerve conduction studies, electromyography, and muscle function evaluation (Daube, 2000). Several causative genes, such as *ALS2*, *DCTN1*, *VAPB*, *ANG*, *TDP-43*, *FUS*, and *SOD1*, have been identified from patients with ALS. Mutations in the representative gene *SOD1* are involved in demyelination, motor neuronal degeneration, and decreased regeneration (McCord, 1994; Andrus et al., 1998; Beers et al., 2006; Yamanaka et al., 2008; Lobsiger et al., 2009). Although the role of SOD1 in motor neurons has been relatively actively studied, little research has been conducted on the role of SOD1 in glial cells. Indeed, overexpression of *SOD1* induces oxidative stress and lipid peroxidation in both motor neurons and glial cells, including Schwann cells (Rosen, 1993; Pasinelli and Brown, 2006).

Previously, it was reported that mice with non-myelinating Schwann cells expressing a point mutation form of *SOD1* (*SOD1* G93A) showed severe motor neuronal degeneration, reduced neuronal regeneration, and accelerated ALS disease pathologies (Lobsiger et al., 2009). Although the relationship between primary cilia in Schwann cells and ALS pathological mechanisms has not yet been elucidated, studies using a mouse model harboring an *SOD1* (G93A) mutation have suggested the involvement of primary cilia in motor neuronal functions (Ma et al., 2011; Osking et al., 2019). Notably, mutated *SOD1* (G93A) increases the expression of *ACTIN* cytoskeleton genes (Perrin et al., 2005; de Oliveira et al., 2013). In addition, *SOD1* (G93A) mutant mice showed increased expression of several Wnt signaling-related genes, including both canonical and non-canonical *Wnts* in the spinal cord (Yu et al., 2013; Table 1). ACTIN-mediated cellular dynamics is important not only for neuronal function but also for ciliary formation and function (Smith et al., 2020), and Wnt signaling is important for initiating myelination (Tawk et al., 2011) and controlling ciliary formation and function (Lee, 2020). Accordingly, studies determining whether primary cilia in Schwann cells are involved in the pathological mechanism of ALS through the regulation of Wnt signaling or ACTIN-mediated cellular function would be of interest.

### Charcot Marie Tooth Disease

Charcot Marie Tooth disease (CMT) is a common hereditary neurological disorder of the PNS, in which the muscles of the hands and feet are gradually lost, making movement difficult (Pareyson and Marchesi, 2009). Among the subtypes of CMT, type 1 is mainly caused by impairment of Schwann cells that play a role in myelination, and the mutations identified in CMT type 1 are found in genes coding for myelination regulators such as PMP22, MPZ, and EGR2 (Hattori et al., 2003). These myelin proteins are expressed in myelinating Schwann cells and are involved in responding to signals that control myelination. For instance, in the process of myelination, MPZ in Schwann cells is essential for responding to cAMP signals derived from neuronal axons (LeBlanc et al., 1992). cAMP signaling plays a critical role in the process of Schwann cell development, including proliferation and myelination (Morgan et al., 1991). Of note, primary cilia are involved in cAMP signaling through adenylate cyclase type III, a representative cilia protein in the nervous system (Wang et al., 2011; Sterpka and Chen, 2018; Table 1). These data suggest that studies investigating the role of primary cilia in myelination-related signal transduction in Schwann cells will help to better understand the mechanisms of demyelinating diseases, such as CMT.

As studies related to the repair mechanism of Schwann cell myelination for the treatment of peripheral degenerative diseases have gained traction, the investigation of the role of Schwann cell cilia in nerve regeneration has also attracted attention (Jessen and Mirsky, 2016). Several recent studies have suggested that primary cilia in Schwann cells are involved in myelination-related signal transduction, such as Shh, Wnt, and Hippo signaling (Tawk et al., 2011; Yoshimura and Takeda, 2012; Morton et al., 2013; Grove et al., 2017; Jiang et al., 2019; Mc Fie et al., 2020; Figure 3 and Table 1). In mouse primary Schwann cells, major Shh components such as SMO and PTCH1 are localized at the base of primary cilia, and these activate Shh signal transduction (Wilson and Stainier, 2010; Yoshimura and Takeda, 2012). Treatment with Shh or SMO agonist results in an increase in myelin segments, suggesting the potential involvement of Schwann cell cilia in regulating myelination (Parmantier et al., 1999; Wilson and Stainier, 2010).

In the MSC80 mouse Schwann cell line, key molecules of canonical Wnt signaling, such as WNT1, LRP6, DSH, GSK3β, β-catenin, and TCF/LEF1, are expressed prior to activation of the myelination regulators PMP22 and MPZ (Tawk et al., 2011). Given that canonical Wnt signaling regulates cilia assembly through WNT3A or cilia disassembly through WNT5A (Lee et al., 2012; Kyun et al., 2020), it is possible to speculate that primary cilia in Schwann cells may be involved in Wnt-mediated myelination. Yes-associated protein (YAP) and transcriptional coactivator with PDZ-binding motif (TAZ), are Hippo signaling regulators, have a key role in the cytoplasm of ciliated cells, but these translocate into the nucleus and induce cell proliferation in the context of ciliogenesis suppression (Rausch and Hansen, 2020). Notably, both YAP and TAZ are expressed in myelinating Schwann cells, but not in non-myelinating Schwann cells (Grove et al., 2017; Jeanette et al., 2021). Thus, this suggests a potential role for primary cilia-mediated Hippo signaling in myelination. More intriguingly, YAP and TAZ play a role in remyelination during Schwann cells-mediated regeneration induced by nerve injury (Grove et al., 2020; Jeanette et al., 2021). Taken together, these data suggest that primary cilia in Schwann cells may be essential in myelination involving several signaling pathways; thus, control of Schwann cell ciliogenesis could be a potential target for the treatment of PNS demyelinating disorders.

### Functional Potential of Schwann Cell Cilia in Nerve Regeneration–Related Signaling

#### Calcium Signaling

Given that the primary cilia in chondrocytes are essential for mechanotransduction via ATP-induced Ca^2+^ signaling, it can be speculated that primary cilia in Schwann cells may regulate mitochondrial mechanotransduction through Ca^2+^ signaling molecules, such as inositol 1.4.5-triphosphate (IP_3_) (Powell et al., 2003; Wann et al., 2012; Phua et al., 2015); IP_3_ and its receptor are also present in Schwann cells, and they play an important role in regulating mitochondrial Ca^2+^ signaling during myelination (Powell et al., 2003; Ino et al., 2015). It is noteworthy that in a Xenopus model, primary cilia are reduced when IP_3_ signaling is inhibited, suggesting that IP_3_ signaling is involved in ciliogenesis (Hatayama et al., 2011). Particularly, in Schwann cells, P2Y2 and P2RY2, which produce IP_3_ and increase the level of mitochondrial Ca^2+^, appear to be associated with ciliogenesis (Weisman et al., 2012; Table 1).

#### Cell Adhesion Molecule-Mediated Signaling

Another notable molecule associated with ciliogenesis of Schwann cells is Polysialic acid (PSA), a homopolymer of α2, 8-linked sialic acid expressed in the nervous systems. PSA is involved in axon guidance and activation of neuronal cell adhesion molecules (NCAMs) (Tang et al., 1994; Lavdas et al., 2006; Papastefanaki et al., 2007). Recent reports have revealed that the PSA-NCAM complex is decreased in the brains of diseased humans and rodents (Varea et al., 2005; Murray et al., 2016), and modified PSA-NCAM complexes can be seen in several neurodegenerative diseases such as AD and PD (Murray et al., 2016). In the PNS, NCAMs are expressed in the membranes of Schwann cells, and these regulate injury-induced remyelination by making a complex with PSA (Angata and Fukuda, 2003; Lavdas et al., 2006; Papastefanaki et al., 2007). Studies using zebrafish models have reported that PSA and NCAMs exist in primary cilia of the CNS; however, this has not yet been investigated in the PNS (Matsumoto et al., 2019). Furthermore, the number of cells expressing NCAMs and PSA was reduced when primary cilia formation was inhibited in the mouse brain (neural stem cells), suggesting an important role of cilia in NCAM-PSA complex-mediated cell proliferation in the CNS (Han et al., 2008; Table 1). Based on these studies conducted in the CNS, we assume that the NCAM-PSA complex present in Schwann cells may also be associated with primary cilia in the PNS.

Several cell adhesion molecules (CAMs), including NCAM, myelin-associated glycoprotein, neuregulin1, neural adhesion molecules (L1), integrin β1, and N-cadherin, are essential for Schwann cell development as well as for cell-cell connection in the PNS (Quarles, 2002; Spiegel and Peles, 2002; Bartsch, 2003; Poliak and Peles, 2003; Michailov et al., 2004; Feltri and Wrabetz, 2005; Taveggia et al., 2005). In particular, CAMs are secreted as autocrine factors from Schwann cells activated by Shh signals during myelination (Parmantier et al., 1999; Hashimoto et al., 2008). Given the important roles of primary cilia for Shh signaling in the control of myelination by glial cells (Yoshimura and Takeda, 2012; Falcon-Urrutia et al., 2015), it is likely that primary cilia in Schwann cells are involved in the secretion of CAMs, including NCAM, to prevent neurodegeneration or to induce nerve regeneration.

#### Neuromuscular Junction-Involved Signaling

Many neurodegenerative diseases of the PNS have features of muscle defects such as muscle weakness, cramps, numbness, and tingling in the hands and feet (Burakgazi and Hoke, 2010; No authors listed, 2010; van Sloten et al., 2011). Typically, these muscle-related symptoms result from impaired innervation at the neuromuscular junction (NMJ), which is regulated not only by muscle cells but also by neurons and Schwann cells (Gordon, 2020). Thus, the sophisticated regulation of cellular communication between these cells is closely related to disease mechanisms in the PNS. In cellular communication, Schwann cells are involved in NMJ formation through TGFβ signaling and signal transmission in the NMJ via Ca^2+^ (Robitaille, 1998; Castonguay and Robitaille, 2001; Sugiura and Lin, 2011; Fuentes-Medel et al., 2012; Kerr et al., 2014; Barik et al., 2016). Under a condition of nerve injury, Schwann cells communicate with neurons by releasing several molecules, such as cytokines, chemokines, and growth factors, to activate neuronal function (Ydens et al., 2013; Wei et al., 2019). These molecules are also involved in the immune responses of Schwann cells that protect neurons from nerve damages and control neurotrophic factors-mediated signaling for nerve regeneration (Agthong et al., 2009; Kidd et al., 2013; Ydens et al., 2013).

#### Neurotrophic Factor-Mediated Signaling

Neurotrophic factors, including brain-derived neurotrophic factor and glial cell line-derived neurotrophic factor, are essential throughout Schwann cell development process, including the migration, proliferation, and (re)myelination stages (Yamauchi et al., 2004; Iwase et al., 2005; Madduri and Gander, 2010). Thus, misregulation of neurotrophic factor-mediated signaling in Schwann cells results in neuronal cell death and defects in axonal regeneration, which in turn leads to the failure of neuronal repair and muscle innervation (Arthur-Farraj et al., 2012; Glat et al., 2016; Guy et al., 2019). Based on the findings that primary cilia are associated with several signal transductions, previous studies have suggested the involvement of primary cilia in neurotrophic factors-dependent signaling in Schwann cells (Yamauchi et al., 2004; Yoshimura and Takeda, 2012). It is noteworthy that Rho GTPases, regulators of ciliogenesis (Kakiuchi et al., 2019), play a key role in neurotrophic factor-mediated signaling in Schwann cells (Yamauchi et al., 2004), and Schwann cells involved in regeneration have primary cilia (Yoshimura and Takeda, 2012). In conclusion, these findings suggest that primary cilia in Schwann cells are essential for signaling to control nerve regeneration (Figure 3). Moreover, the regulatory mechanisms for Schwann cell ciliogenesis could be a potential target for PNS regeneration therapy.

## Conclusion and Perspective

Primary cilia play a variety of essential roles in the development of the nervous system and are implicated in several neurological diseases. Many studies focusing on the function of neuronal primary cilia have provided insights into the importance of targeting cilia-mediated signaling for the treatment of neurological diseases (Kirschen and Xiong, 2017). However, as these studies were focused on neurons, our understanding of the mechanisms of neurological diseases associated with damage to other types of cells, such as glial cells, is limited. There is growing evidence that primary cilia-mediated signaling in glial cells may be closely linked to the pathogenesis of several neurological diseases (Sterpka and Chen, 2018). In this review, we have presented the latest findings related to glial primary cilia to facilitate understanding of the cilia-mediated function in each glial cell type. In particular, we sought to understand the relationship between the signaling pathways regulated by disease-causing genes and primary cilia in glial cells in each neurodegenerative disease.

Inhibition of the progression of chronic systemic inflammation has been proposed as an approach for the treatment of neurodegenerative diseases (Norden et al., 2015). From this perspective, NF-κB is considered a major target, and Aβ peptide, a major causative molecule in AD, is implicated in the regulation of inflammatory responses through NF-κB activation (Liu et al., 2014). In addition, several related studies have reported that inhibition of NF-κB signaling reduces Aβ-induced neurotoxicity in neuron and glial cells (Yan et al., 2020; Zamani et al., 2020). It is also notable that Aβ peptide induces aberrant cilia formation by interfering with Shh signaling (Vorobyeva and Saunders, 2018). Moreover, LRRK2, a causative molecule in PD, plays an important role in ciliogenesis by activating ciliary regulators, such as RAB8A and RAB10 (Steger et al., 2016, 2017; Alessi and Sammler, 2018), and in inflammatory responses by regulating NF-κB signaling (Kim et al., 2012; Lopez de Maturana et al., 2016). Therefore, the regulatory mechanism of cilia formation may be a novel target for treating neurodegenerative diseases by controlling NF-κB-mediated inflammation. In conclusion, the potential role of astrocyte cilia in the regulation of inflammation in the nervous system implies that the regulatory mechanisms for ciliogenesis in astrocytes could be a novel target for the treatment of neurodegenerative diseases such as AD, PD, and HD. Moreover, screening of anti-inflammatory drugs and small molecules targeting the primary cilia will help discover and develop therapeutic agents for these diseases in the future.

It has been previously suggested that regulation of microtubule dynamics is essential for the development of glial cells, such as OLGs and Schwann cells in the CNS and PNS, respectively (Richter-Landsberg, 2008; Bauer et al., 2009; Yokota et al., 2009; Eom et al., 2011). In particular, OLGs that undergo morphological changes from OPCs during differentiation/maturation for myelination (Miller, 2002) require adequate microtubule dynamics. The regulation of microtubule (de)polymerization and stabilization is critical for interactions of OLGs with neuronal axons (Fu et al., 2019; Lee and Hur, 2020), and the assembled microtubules are important for intracellular transportation of myelin-related proteins, including MBP (Smith, 2004). Histone deacetylases, including SIRT2 and HDAC6, are well known ciliary proteins that modulate cilia disassembly (Pugacheva et al., 2007; Zhou et al., 2014; Lim et al., 2020), and recently, they have been shown to be involved in the microtubules-mediated remodeling of OLGs (Hubbert et al., 2002; Southwood et al., 2007; Noack et al., 2014). In addition, a study has revealed that stathmin, a negative regulator of microtubule dynamics (Ozon et al., 2002), interferes with OLG differentiation, leading to demyelinating diseases such as MS (Liu et al., 2005). In Schwann cells, microtubule-associated protein 1 B binds to α1-syntrophin (Fuhrmann-Stroissnigg et al., 2012), a member of the adaptors acting on signal transduction, or microtubules (Togel et al., 1998) and regulates microtubule dynamics for Schwann cell migration (Bouquet et al., 2007). Although studies demonstrating a direct association of misregulation of microtubule dynamics in Schwann cells with neurodegenerative diseases are lacking, these previous findings suggest that adequate control of primary cilia-involved microtubule dynamics in glial cells can be a target for the treatment of demyelinating diseases.

Since impairment of autophagy mechanism has been frequently reported as a major cause in neurodegenerative disorders (Ravikumar et al., 2005; Lee et al., 2010; Nixon and Yang, 2011; Gomez-Suaga et al., 2012; MacLeod et al., 2013), autophagy regulation may be a target for the treatment of neurological diseases involving glial cells. Mutations in *PRESENILIN 1*, a major genetic factor in AD, disrupt autolysosomal proteolysis and accelerate the onset and severity of AD (Lee et al., 2010; Nixon and Yang, 2011). Mutations in *LRRK2* leading to endosomal-lysosomal trafficking, lysosomal pH and calcium regulation are implicated in PD pathogenesis (Gomez-Suaga et al., 2012; MacLeod et al., 2013). Autophagosomal accumulation induced by the mutated dynein complex increases aggregation-prone proteins in ALS (Ravikumar et al., 2005). Similar to that in other types of cells, autophagy is critical for the developmental processes of OLG and Schwann cells, including for myelination (Jang et al., 2015; Bankston et al., 2019). Indeed, disruption of autophagy-related regulators or signaling affects myelin regulators, such as MBP, resulting in impaired myelination (Dello Russo et al., 2013). Additionally, proper autophagy is essential for removing myelin debris derived from injury to Schwann cells (Gomez-Sanchez et al., 2015; Jang et al., 2015). During ciliogenesis, the reciprocal interactions between ciliary proteins and autophagy molecules are tightly controlled (Pampliega et al., 2013; Tang et al., 2013; Orhon et al., 2015). Hence, these studies suggest that primary cilia of glial cells are involved in the regulation of autophagy during the process of myelination and that the cilia-mediated autophagy mechanism may be a novel target for the treatment of myelination-related diseases. Taken together, studies on the regulatory mechanisms and functions of primary cilia in glial cells will pave a new path for the development of novel paradigm therapies as well as provide a comprehensive understanding of the pathological mechanisms of neurodegenerative diseases in both the CNS and PNS.

## Author Contributions

SK, HJ, and JL produced ideas for review contents and wrote the manuscript. All authors contributed to the article and approved the submitted version.

## Conflict of Interest

The authors declare that the research was conducted in the absence of any commercial or financial relationships that could be construed as a potential conflict of interest.

## Publisher’s Note

All claims expressed in this article are solely those of the authors and do not necessarily represent those of their affiliated organizations, or those of the publisher, the editors and the reviewers. Any product that may be evaluated in this article, or claim that may be made by its manufacturer, is not guaranteed or endorsed by the publisher.
